# An epidemiological perspective on bovine tuberculosis spotlighting facts and dilemmas in Iran, a historically zebu-dominant farming country

**Published:** 2013-03

**Authors:** Keyvan Tadayon, Nader Mosavari, Mohammad Mehdi Feizabadi

**Affiliations:** 1 Department of Veterinary Aerobic Bacterial Research & Vaccine Production, Razi Vaccine and Serum Research Institute, Karaj 3197619751, Iran; 2 PPD Tuberculin Department, Razi Vaccine & Serum Research Institute, Karaj, Iran; 3 Department of Microbiology, School of Medicine, Tehran University of Medical Sciences, Tehran, Iran

## Abstract

For the whole 20th century, bovine tuberculosis (BTB) challenged the international community efforts to control this zoonotic disease. Asia and Africa accommodate the largest BTB-infected zebu cattle in the world. Similar to other few Asian nations, Iran has been actively running its BTB-control plan for the last four decades. BTB however, is still a number-one health concern for Iranian veterinary practitioners and also farmers across the country. Why is that? Here we have addressed this question in the light of most recent epidemiological data as well as microbiology and molecular biology observations.

## Facts and dilemmas in the history of bovine tuberculosis in Persia

### Iran, a historically zebu-farming country in Asia

**Fig. 1:**
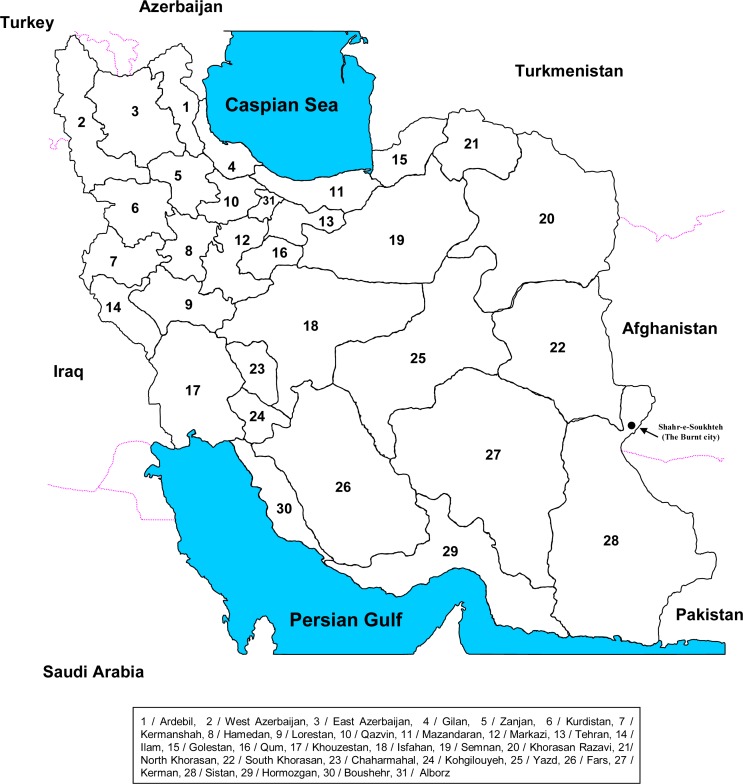
Iran, a country in the Middle East. Shahr-e-Shoukhteh (the Burnt city) is marked.

Iran (formerly known as Persia) has some of the oldest historical records on cattle farming in the world. The Zagros mountain chain in the west of Iran, a part of the Fertile Crescent with its ideal climate for animal farming, and the historic city of Soukhteh in Eastern Iran, have been recognised as birthplaces for cattle domestication (Fig. 1) (1).

Since 2010, Iran comprises 31 provincial regions which have a diverse range of climatic conditions from the tropical areas on the coast of the Persian Gulf to the deserts and arid lands on the Eastern half of the country (Fig. 1). There are also wet and colder areas with forests in the North and West. The temperature range in some locations in Iran can be as great as 50°C. In some geographical areas of Iran, especially in the drier locations, cattle farmers have to buy forage for their animals, and this could originate from neighbourhood cities or even from the other side of the country.

The national cattle herd size in Iran in 2010 was about 8.5 million head (2). As with many other Asian countries; zebu cattle characterised by their broad horns and often hump including 7 identifiable local breeds Bami (Dashtiari), Golpayegani, Kurdi, Mazandarani (Gilani), Sarabi (Ardebili), Sistani and Talesh have been farmed for a long time in Iran.

In the 1930s, the first attempts at modernisation of animal farming in Iran were initiated. In March 1935 a French veterinarian, Dr Wactan, employed by the former Agriculture Office, imported 16 bovids of Simmental, Tarentaise and Schwyz breeds from France. In 1936, five more Schwyz beasts were imported from the former USSR and all these animals were kept on the government farm of Heidar-Abad (Alborz province) in the suburbs of Tehran (A.A.,Gharahbaghi, personal communication). Since the 1960s, when Holstein-Friesian (HF) bovids were imported into Iran for the first time, HF cattle gradually became the predominant and favourite breed for Iranian farmers and were imported from several countries including USA and Canada.

## Old and contemporary biogeography of BTB in Iran

Records of BTB in Iran date back to the first half of the last century. In 1931, Carpantier, a French veterinarian employed by the Iranian army was the first to report BTB in zebu cattle slaughtered at Tehran old abattoir. Later in 1936 and 1937, these observations were added to by local veterinary officers from other Iranian cities. Some of the formalin-preserved BTB specimens collected at the time are still present in the veterinary laboratories of the Iranian Army in Tehran.

An increasing number of the animals on Heidar Abad State farm were reported tuberculous in the early years after their importation and by 1947, 70 out of the 75 animals now on this farm were confirmed with BTB (3).

Furthermore, in 1945 BTB was confirmed in the cattle herd of the Abadan National Oil Company, the second modern government cattle farm in Iran. The cattle of this herd were also European breeds that had been imported from abroad (3, 4).

It is postulated that cattle imported into Iran from France in 1935 and probably from the UK in 1945 were responsible for the introduction European strains of *M. bovis*. Similarly, an epidemic of BTB was reported in the imported cattle at the second government farm of the Abadan National Oil Company in 1945, which had probably imported cattle from the UK (3).

In September 1948, in an official report to the Iranian Agriculture Minister at the time, Louise Paul Delpi, pointed out that all the imported cattle from France to Heidar Abad farm were tuberculinated and passed this test in France just before their transport to Iran.

**Fig. 2:**
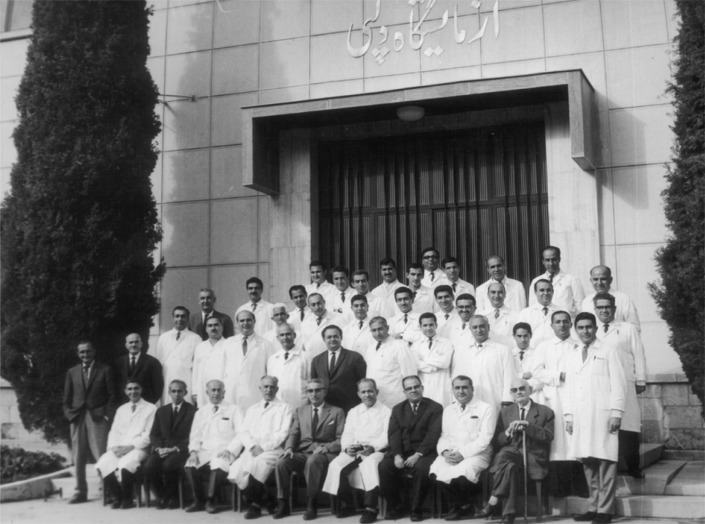
Fathollah Entesar (third from left, seated) with his colleagues at the RVSRI silver anniversary celebrating 25 years of progress in research and production of biologicals, Hessarak, Karaj, 1956. To attend the reunion, Louis P. Delpy (center, seated) flanked by Hossein Mirshamsi (right) and Morteza Kaveh (left) traveled from France to Iran, where he served as RVSRI general director for 20 years (1930-1950). Photograph courtesy of Dr Mahmoud Ardehali (right, standing in front).

In 1945, Fathollah Entesar from the Razi Institute successfully isolated *M. bovis* from a tuberculous cow owned by a farmer from Esmail Abad village in Alborz province, and showed its pathogenicity in laboratory animals (F. Entesar, personal communication) (Fig. 2).

From 1941 onwards, along with increases in the importation of cattle and the commissioning of new cattle farms, particularly in the suburbs of Tehran, more and more breakdowns of BTB were reported in Tehran. This was believed to be partly due to the ignorance of farmers about the basic hygiene standards required for intensive cattle farming. On the other hand, at Razi Institute in the Eastern suburbs of Tehran, tuberculous lesions were regularly reported in cattle from local breeds which had been brought by the Institute from different Iranian provinces in order to produce Rinderpest vaccine against cattle plague which posed a big threat to bovids in Iran in 1940s (3, 4).

**Fig. 3:**
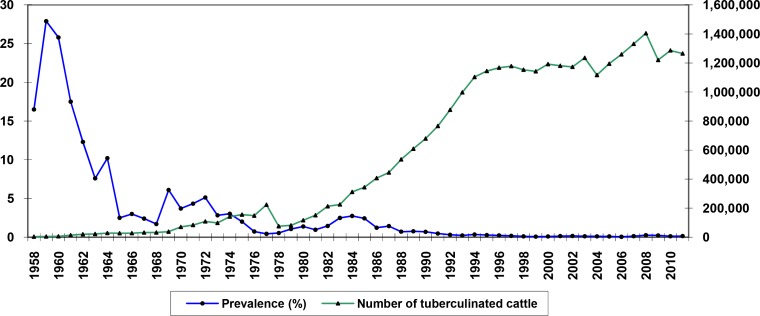
Extension of the test and slaughter program and frequency of tuberculin positive cattle in Iran, 1958-2011.

In 1952 the first serious attempt to comprehend the epidemiology of BTB in Iran was made. This research was conducted under Jurgensen, a veterinarian adviser from FAO, in association with IVO and the Razi Institute. In 1967, legislation for the test-and-slaughter program that was compulsory for modern (European style) and semi-modern cattle farms, as well as traditional farms located within their perimeters was brought in. From 1967 until 1977 the test-and-slaughter program was noticeably expanded and apparently lowered the prevalence of BTB in Iran (Fig. 3). In 1969, epidemics of Rinderpest (cattle plague) struck Iran once again and led to a stalling of all BTB control activities while the IVO staffs were engaged in dealing with this epidemic.

### BTB in human

While there are a number workers reporting post-vaccinal complications after BCG-vaccination of children and adults in Iran (5–7), only few published studies have reported infection of human cases with *M. bovis* in this country (8). In a study searching for *M. bovis* in 1952, of 30 human tuberculosis cases isolated in the Shah Abad Sanatorium in Tehran, no *M. bovis* infection was detected. In 2007 Velayati reported infection of two children in a family with a wild strain of *M. bovis* (9). In 2011, Tadayon and co-workers genotyped two *M. bovis* isolates collected from tuberculous patients in Markazi province among which one hold a BCG-like spoligotype (SB0120) and the second isolate represented a new spoligotype (SB2182) (Tadayon, unpublished data).

**Fig. 4:**
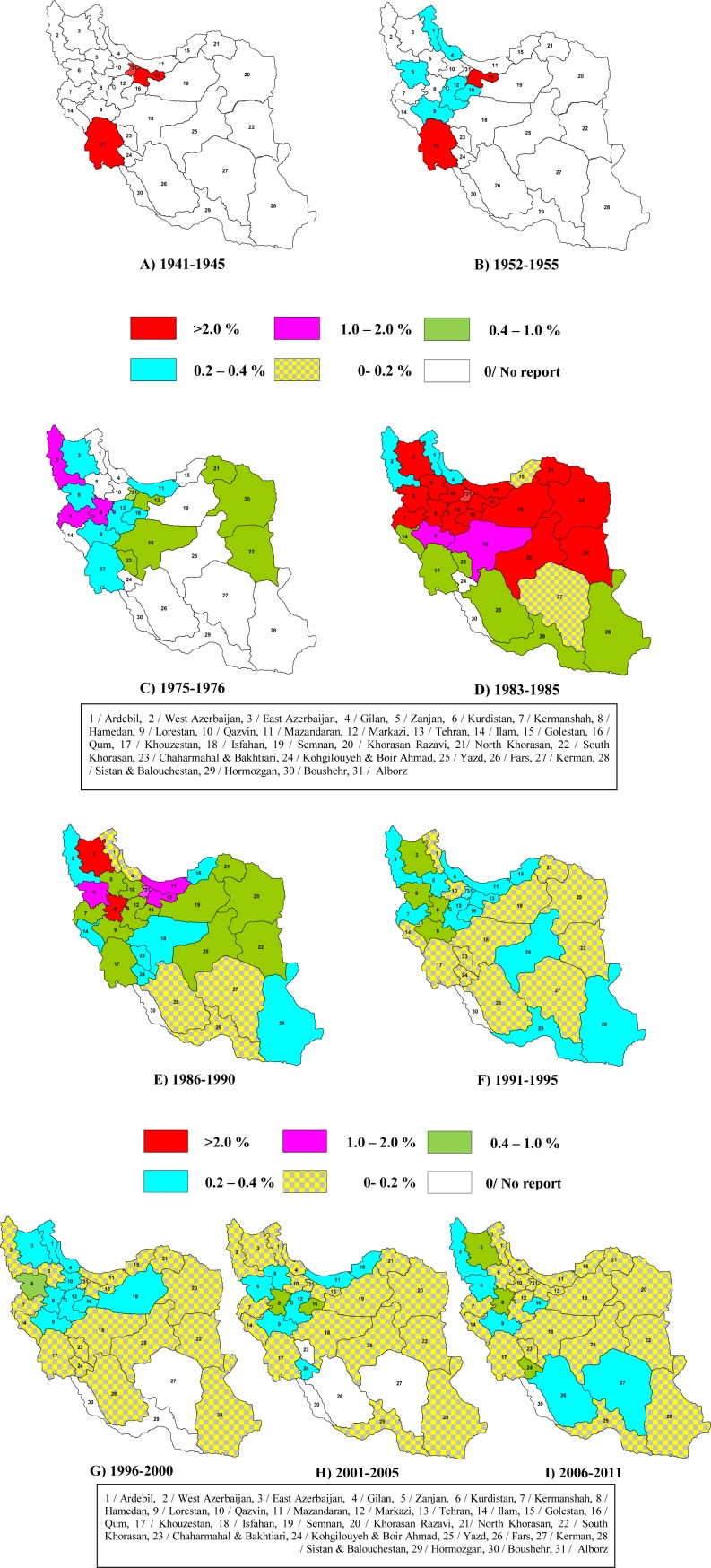
Prevalence of BTB in the 31 administrational provinces of Iran, 1941-2011.

The epidemiology of BTB over the last 80 years in the Iranian cattle herd can be summarised as follows:

1. BTB has been found repeatedly in cattle of local breeds in Iran. The infection in local breeds does appear to be less frequent, and with a sporadic nature compared to that in European bovids.
2. It is very likely that BTB had a broader spatial distribution than that reported in IVO records of the 1940s and 1950s. The results of geographically-limited tuberculinations in the mid 1950s identified BTB in several Northern and Western provinces of Iran. However, later tuberculinations by IVO after 1980s detected the disease in the vast majority of Iranian provinces, indicating that BTB was epidemiologically endemic throughout much of Iran (Fig. 4). Whether this broad distribution was a reflection of a historical pattern or was a recent consequence of the massive expansion in cattle farming over recent years, is not clear from the historical records.
3. Poor health measures in the husbandry of imported European cattle in 1940s and 1950s may have led to extensive epidemics of BTB particularly in the Tehran suburbs where most of the newly established farms were located. Over these years the highest ever prevalences of BTB were in farms with European cattle (28%).
4. The 4-year lapse in the test-and-slaughter program from 1978 to 1981 due to the nation-wide strikes and establishment of the Islamic Republic of Iran and declaration of war on Iran by Iraq resulted in a rise in the prevalence of BTB from 0.43% in 1977 to 2.74% in 1984. Within a few years of the program starting to operate again, this figure re-commenced its downward trend and is still decreasing (Fig. 4). This ‘intervention’ suggests that the test-and-slaughter program had a significant effect on reducing the incidence of BTB.
5. The declining annual reports of BTB breakdowns in Iran has apparently been the result of the test-and-slaughter program but the program has not yet eradicated the disease from any Iranian province. Almost all IVO regional offices in Iran continue to report the BTB every year. The status of BTB in Southern provinces is noteworthy. Boushehr and Hormozgan, which participate in a test-and-slaughter program similar to other Iranian provinces, still report few outbreaks of BTB in some years thus, they are not considered BTB-free. These provinces have low cattle densities and they are not important farming zones compared to other parts of Iran. Further, these two provinces are coastal regions located on the Persian Gulf and thus geographically isolated. Moreover, the neighbouring provinces of Fars, Khouzestan, Kerman and Sistan also commonly have low prevalence of the disease (Fig. 4).
6. Almost a quarter of Iran, predominantly the North Western provinces, continues to be a major stronghold of BTB. The test-and-slaughter program has improved the status of BTB in this part of Iran but it still reports the highest rates of the disease.

## The test-and-slaughter program in Iran: pitfalls and impediments

The national test-and-slaughter program in Iran is based on the single comparative intradermal tuberculin test (SICTT) using locally-prepared tuberculins. The bovine tuberculin and avian tuberculins are both provided by the State-run Razi Vaccine and Serum Research Institute (RVSRI). The avian tuberculin is prepared from the standard strain of *M. avium* D4 and the bovine tuberculin is made from *M. bovis* AN5.

Despite the successes of the test-and-slaughter program in Iran, it suffers from a number of weaknesses:

### Partial coverage of national herd

It is estimated that almost 10% of the national cattle herd in Iran is regularly tuberculin tested by the program.

### Not all the detected reactors are culled

Complete culling of all the identified reactors within the legal deadline of 20 days is not normally achieved leading to a remaining portion of potentially infectious cattle on their original farms, probably in contact with other cattle. The IVO records explain that in the majority of cases farmers are not satisfied with the compensation they would receive for their reactor animals. Consequently, some animals may be slaughtered and sold illegally before the deadline or farmers may have tried to hide or sell them.

### Focus on European (HF) cattle breeds

Farmers in rural areas of Iran tend to farm local, zebu breeds of cattle, they have small numbers of animals in their herds and grazing on pasture is typical. The density of local breeds in rural areas is lower than on the intensive farms of urbanised areas. On the other hand poorer health measures are likely to be in place and access to veterinary services is more difficult. Abattoir observations and the test-and-slaughter program results gathered over several decades show a higher rate of BTB in European breeds of cattle compared to the zebu local breeds (3). This has been noted in other countries as well (10, 11). The higher market values of European breeds as well as better production indices and adaptability to intensive farming are economically important characteristics of the European breeds. Thus, a larger number of these breeds are normally farmed on the intensive farms compared to the traditional farms in rural areas of Iran.

In 1993 a study by Khaladj from IVO on BTB in the traditional cattle farms in Iran showed a 0.14% prevalence of the disease in cattle on these farms (3). In this research 49,414 cattle of local breeds from 627 villages throughout Iran were tuberculin tested. This research showed the necessity for further epidemiological study of BTB in local breeds of cattle in Iran and in understanding the link between BTB in these bovids and European cattle.

### Disseminated BTB

Cattle carcasses with disseminated disease are not normally reported from countries with efficient test-and-slaughter programs (12). Indeed, a common reason for condemnation of cattle carcasses in Iranian abattoirs is the finding of disseminated BTB. Of particular concern is that this form of the disease is not exclusively found in cattle with no history of tuberculin testing, but is also seen in tuberculinated reactors. In 1993, a total of 1,268,140 cattle were slaughtered in Iran among which 906 (0.07%) carcasses were totally and 8,175 (0.64%) were partially condemned due to BTB. Of the carcasses that were totally condemned 216 (24%) had been tuberculinated and this figure for partially condemned carcases was 1,493 (18%) (3). Further, IVO records for West Azerbaijan showed on average 20% of the total condemned cattle carcasses in the six year period from 1988 to 1994 were reported as having tuberculous lesions. These records support the concern that the interval between successive tuberculin tests is sufficiently long for BTB infection to progress to disseminated disease in some animals. In Iran, performing a second tuberculin test within a year is limited to a very few areas, most particularly to Tehran.

### Lack of systematic data collection

Cattle of local breeds do not usually have any individual identifiers, such as ear-tags, while in contrast almost all European breed animals bear a means of identification that is typically an ear tag. Hence, keeping records and tracing back the origin of animals by IVO staff in abattoirs is difficult or sometimes impractical unless extra arrangements are in place prior to the slaughter. This makes it hard to trace the origin of infected animals to identify infected herds and so assess the performance of the test-and-slaughter program. In 2008, with the implementation of the Geographical Information System (GIS), the IVO initiated its first nation-wide system for collection of epidemiological data on animal infectious diseases (IVO, unpublished data).

### Lack of reference mycobacteriology laboratory

With more than 40 years of operating the test-and-slaughter program, there are only a handful of researchers conducting microbiological studies on BTB in Iran. The main reason is a lack of appropriate laboratory infrastructure. This dearth is not specific to Iran but is common over the whole of the Middle East. Occasional microbiology services for the IVO are provided by the Pasteur and Razi Institutes in Tehran and Karaj respectively, but these are not sufficient. The Razi Institute is mainly involved in the production of PPD tuberculins required for the test-and-slaughter program, and although it does provide a veterinary mycobacteriology diagnostic service there is a desperate need for IVO to establish a modern veterinary laboratory which would operate as an accredited reference facility.

### Inconclusive reactions in the tuberculin test

Cattle without a conclusive test result may or may not be infected with *M. tuberculosis* complex pathogens (most probably *M. bovis*). They may perhaps alternatively be infected with environmental mycobacteria that induce reaction against tuberculin (13–15). The epidemiology of environmental mycobacteria in cattle does not follow the same pattern as of *M. bovis*, as culling these animals would not have a major impact on the abundance of these mycobacterial species in the environment. A study by Khavari Khorasani from IVO showed that environmental mycobacteria were able to induce a positive tuberculin reaction in Iranian cattle and even to cause severe tuberculous-like lesions (4). In the IVO study the pathological specimens (mediastinal and head lymph nodes plus lungs) from 269 tuberculin positive bovids from East Azerbaijan and Khorasan were subjected to bacterial culture. In total, 40 isolates were culture positive of which 20 were identified as *M. bovis*, and the others identified as *M. chelonei* (1 isolate), *M. flavescence* (9 isolates), *M. szulgi* (2 isolates), *M. terrae* (1 isolates), fast growing mycobacteria (5 isolates) and two unidentified isolates. Interestingly, all but four of these beasts had visible lesions in the carcass, which resulted in partial or total condemnation of their carcasses at abattoir. Khavari concluded that environmental mycobacteria in Iran are probably able to produce extensive tuberculous-like lesions in cattle.

The IVO records also report infection of cattle with parasitic diseases such as Fasciolosis (caused by *Fasciola hepatica*) and Schistosomiasis (caused by *Schistosoma bovis*) can raise false positive reactions in the tuberculin test (3, 16, 17). This observation might explain why non-tuberculous lesions in the liver are sometimes reported in tuberculin positive cattle in Iran.

### No herd-depopulation strategy

Any test-and-slaughter program is most successful when all the susceptible animals in a herd with a positive tuberculin test reactor are slaughtered. This measure removes any possibility of disease transmission from an infected animal to other, as yet uninfected and so maintaining infection in the herd. This approach has been successfully used in countries such as Australia where eradication of BTB has been achieved. In the UK, depopulation of cattle farms, either partially or totally, is undertaken as often as necessary where chronic BTB herd breakdowns are encountered. In Iran this plan has not been instituted due to funding difficulties.

### Animal reservoirs

In some countries such as the UK and Ireland there is significant involvement of animal reservoirs in the epidemiology of *M. bovis* and this has impeded the eradication of BTB. In Iran, in a study by IVO, *M. tuberculosis* complex bacteria were isolated from three rats that were trapped on cattle farms with a record of BTB in the province of Tehran (18). A similar study in Isfahan resulted in the isolation of acid-fast bacteria from a few trapped house mice on cattle farms with a history of BTB. An isolate collected from one of these mice was identified as *M. bovis*. Tadayon and co-workers showed this isolate carried spoligotype (SB1168) and VNTR type (6554334) of one of the three most common clones of *M. bovis* in Iran. Whether transmission was from cattle to these mice or vice versa is unknown. Buffalos with a population of 650,000 heads (2010) are also farmed in some parts of Iran. West and East Azerbaijans, Khouzestan and Ardebil provinces accommodate 90% of the national buffalo herd. These provinces are also important cattle farming regions. In West and East Azerbaijan many farmers exercise mixed-farming of cattle and buffalo on their farms and pastures. Buffalos are not normally tuberculinated in Iran. Tadayon and co-workers reported collection of a single *M. tuberculosis* complex bacterium from bacterial cultures of a buffalo carcass slaughtered at Urmia, the central city of West Azerbaijan province. Their work included lymph node specimens from 140 slaughtered buffalos representing majority of province herds (19). This isolate was later identified as *M. tuberculosis* (Tadayon, unpublished data). However, until now no microbiologically-confirmed case of buffalo infection with *M. bovis* has ever been observed in Iran. Sheep and goat (with 54 and 25.7 million heads in 2010 respectively) are also frequently farmed in Iran. The latest records of IVO show that tuberculosis does occasionally affect goats. In 2003 *M. tuberculosis* complex spp. had been isolated from four goats in Kurdistan in North West of Iran. There are no confirmed reports of BTB in sheep in Iran. In 2003, abattoir specimens from a sheep carcass with typical tuberculosis lesions were processed for bacterial isolation. The culture on specific media for *M. tuberculosis* complex mycobacteria and incubation of the organism at 37°C successfully yielded bacterial growth. Nevertheless, the very weak bacterial growth hindered further molecular investigations in order to identify this isolate. In 2009, bacterial culture of lymph nodes from a follow deer in Hoveizeh, Khouzestan resulted in collection of a *M. tuberculosis* isolate that were later understood to hold a specific spoligotype (SIT587). In all, the role of reservoir animal(s) in epidemiology of BTB in Iran is as yet unknown and requires further studies.

### Uncontrolled cattle movements

There are currently some limitations on cattle movements between different cities in Iran such as permanent IVO check points on major roads, but these are not adequate. Indeed the present farming system of Iran, particularly in rural regions, is hardly amenable to such restrictions unless new legislation is brought in to restrict cattle movements in order to augment the test-and-slaughter program.

### Molecular epidemiology of BTB in Iran

**Fig. 5:**
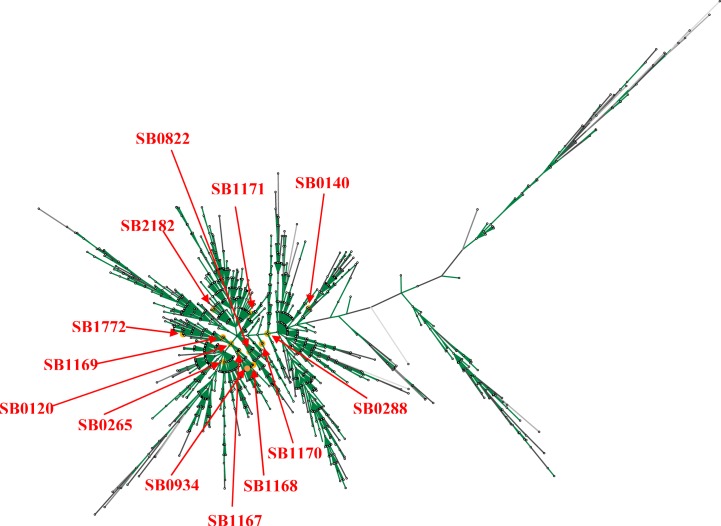
Maximum parsimony tree of 1,476 reported spoligotypes from around the world as of Oct 2012 deposited in the M. bovis spoligotyping database (http://www.mbovis.org/spoligodatabase/user-database-output.php). The spoligotypes found in Iran are indicated

**Fig. 6:**
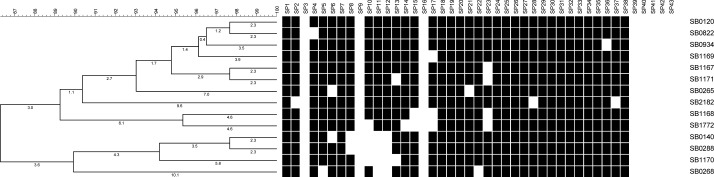
Thirteen spoligotypes of Iranian isolates found up to now with Dendrogram of profile relatedness by Dice and UPGMA made by BioNumerics software (version 6.1; Applied Maths, Belgium). The *M. bovis* AN5 spoligotype (SB0268) is included for comparison.

Feizabadi was first to employ molecular techniques to understand the epidemiology of BTB in Iran. Initial molecular genotyping of *M. bovis* reported that 6 of 7 *M. bovis* isolates carried a single copy of IS*6110* on the discriminative 1.9 kb restriction fragment and one isolate had three copies of IS*6110* (20, 21). Cousins also reported three spoligotypes SB0140, SB0265, and SB0822 from Iran. Tadayon et al. (2008) have more recently genotyped a large collection of mycobacterial isolates (N = 133) collected from abattoir bovine specimens (22). This work confirmed *M. bovis* as the single principal cause of BTB in Iran. None of the studied isolates were *M. bovis* BCG and this categorically ruled out concerns over interpretation of the tuberculin test in cattle due to unauthorized vaccination of cattle with BCG vaccine. Spoligotyping revealed a genetically homogeneous population of *M. bovis*. Only eight, highly similar, spoligotypes were identified with five of them not previously reported elsewhere (SB1167, SB1168, SB1169, SB1170, SB1171; Fig. 5). The ancestral (possibly European) BCG-like spoligotype, SB0120, was the most prevalent (41%) along with two similar patterns, SB1167 (39%) and SB1168 (17%) which were unique to Iran (Fig. 6). VNTR typing on the other hand, recognized 23 types and again revealed a homogeneous population with only a few predominant patterns. In general, the same genotypes were found in both European and zebu breeds in Iran (22). Considering the long history of cattle domestication, the geographical breadth of Iran, the relatively recent importation of European breeds, the expanding herd size and the ongoing test-and slaughter program, the homogeneity of the *M. bovis* isolates from the cattle population was unexpected. Tadayon believes this lack of diversity is probably a reflection of the significant increase in population of, BTB susceptible, Holstein-Friesian (HF) cattle in the last fifty years. Tadayon et al. (2008) speculates that the absence of adequate initial BTB control measures could have led to the rapid clonal expansion of an SB0120 strain.

These findings have been confirmed by a subsequent study (23) in which RFLP genotyping was employed to analyze 123 isolates collected from slaughtered tuberculosis-suspect cattle and one clinically asymptomatic buffalo. DNAs from the isolatse were subjected to *Pvu* II and *Alu* I-digestion and RFLP-PGRS typing. All of these methods detected a visibly homogenous population in which variants were generally only detected in a few isolates. Only *Alu* I-based RFLP was able to clearly differentiate between BCG and field strains of *M. bovis*. As in previous reports, these findings seem to reflect the recent dispersal of a few strain(s) in Iran following the substantial expansion of HF cattle over the last few decades.

## *Mycobacterium bovis* in Iran, exotic or indigenous?

At present, BTB is endemic across much of Iran although the current prevalence of infection is low (Fig. 3). The recent molecular epidemiology findings for *M. bovis* in Iran, raises the interesting question as to the origins of *M. bovis* strains currently found in Iran. These strains might have been indigenous, exotic or a mixture.

### Strain exchange between neighboring countries

A link between Iranian strains and those of its neighboring countries (i.e. Turkmenistan, Azerbaijan, Armenia, Turkey, Iraq, Pakistan and Afghanistan) is likely. Unfortunately, there is a lack of typing studies on *M. bovis* isolates from cattle in these countries. The closest spoligotype between Turkey (SB1198) and Iran (SB0288) has deletion of spacers 3, 8, 9, 10, 11, 12, 16, and 39–43, although spacer 23 is exclusively deleted in the Turkish spoligotypes. It is interesting that SB0288 has been reported from West Azerbaijan which is actually the bordering province between Iran and Turkey. However, there is no VNTR data for the Turkish isolates for further analysis.

### Iran has systematically imported live cattle in the past

Various studies have identified the role that imported cattle have played in the spread of *M. bovis* as a consequence of European colonization (24, 25).

The predominant Iranian spoligotype (SB0120) has been found as a common strain type in most European countries and countries that imported European cattle (26) (Fig. 6). Commonality of spoligotype may not necessarily mean common origin. It is possible that some of these common patterns are the result of homoplasy rather than common epidemiology.

### BTB in indigenous (zebu) and exotic (HF) cattle of Iran

There are reasons to believe that zebu cattle and West African Dama beasts are more resistant than Jersey/Guernsey cattle to BTB (27). An abattoir study in Chad revealed that Mbororo cattle are more likely to develop BTB than Arab zebu bovids and this observation has also been reported from Cameroon (28). In 1993 a study by Khaladj found a 0.14% prevalence of BTB in zebu bovids compared with 0.22% in HF cattle (4). One explanation for this feature is that the local breeds of cattle have a level of immunity due to their long exposure to the pathogen but the exotic HF cattle are immunologically naive having had no previous exposure to the pathogen. The low incidence of BTB in zebu, suggests that zebu are not a significant reservoir of infection for HF cattle.

Of the fourteen spoligotypes reported to date in Iran, seven (SB1167, SB1168, SB1169, SB1170, SB1171, SB1772, SB2182) have only been reported from Iran and these account for over 60% of all the typed isolates until now (Fig. 6). Some of the Iranian spoligotypes have been reported from elsewhere in the world although in one instance (SB0265) this was shown to be a similarity by chance (20). The similarity of other, usually rarer, Iranian spoligotypes to isolates from elsewhere can probably be accounted for by homoplasy (29), which seems likely given the simplicity of many of the Iranian profiles. The predominant Iranian strain type was the SB0120 and of 55 isolates with this profile, 42 had a common VNTR profile, suggesting that these isolates, or perhaps one of the related isolates, would be the ancestral strain. It has been suggested that spoligotypes change over timescales of 11 (30) to 50 years (30). The postulated expansion over nearly 50 years of the Iranian *M. bovis* population and the generation over this period of 2 or 3 sequential spoligotype changes is clearly compatible with these timescales. The absence of geographical regionalization of strains in Iran may also reflect the shorter timescale of events than in the UK with insufficient time for the significant diversification of new strains. The similarities of the Iranian isolates to each other at very high level suggest a single, common ancestral strain of the SB0120 spoligotype. Whether such an ancestral strain originated in Iran or had been imported into Iran could only be established through further genotypic comparison of Iranian SB0120 isolates with others from neighboring countries and from around the world.

### Cattle movements and herd size

Every year, thousands of cattle are moved between farms, animal auctions or shows and abattoirs in Iran. This practice can contribute to the spread of *M. bovis* strains and would also reduce strain regionalization, as has been seen in Cameroon (31). In consequence, this rapid clonal expansion of *M. bovis* would lead to the homogeneous population structure now seen. Therefore, it is postulated that new Iranian-based strains will evolve.

## The impact of the test-and-slaughter program

The Iranian 40-year test-and-slaughter program has probably contributed to the clonality of the *M. bovis* population in Iran. Recent extensive studies in Europe have shown that test-and-slaughter programs can dramatically reduce prevalence of *M. bovis* infected cattle herds and as a consequence the *M. bovis* population and its diversity. This is highly likely to have led to geographical localization of *M. bovis* strains (25).

### Animal reservoirs

Molecular genetics studies on *M. bovis* isolates from badgers in the UK and Republic of Ireland, possums in New Zealand and wild boars in Spain have revealed that such isolates carry specific genotypes that distinguish them from cattle isolates (32). Therefore, transmission of *M. bovis* between wild animals and cattle, if it happens, can be shown by similarity of genotypes between them. In Tadayon's study, one genotyped *M. bovis* isolate was collected from a house mouse trapped in a cattle farm in Isfahan. This farm had a history of BTB. The spoligotype (SB1168) and VNTR type (6554334) of this isolate showed it to be one of the three common clones of *M. bovis* in Iran. Overall, little is known in Iran about other reservoirs of *M. bovis* and the findings of recent work confirm there is a need for further epidemiological studies on *M. bovis* in wild, and domestic, animals of Iran and their potential role as hosts for this pathogen.

## More recent findings

Using deletion analysis on over a thousand *M. bovis* isolates from across the world, Smith and co-workers recently identified a globally important clonal complex of *M. bovis* characterized by deletion of a genomic locus named RDEu1. While this group of *M. bovis* isolates are frequently found in UK, Ireland and their former commercial partners including South Africa, USA and Australia and even Kazakhstan but they have not been reported from Iran until now (33). Similarly, African 1 and African 2 clones of *M. bovis* seem that are frequently observed in Africa are not presents in Iran (34). Whether the most recently-found *M. bovis* clone (European 2) is also not present in Iran, further work is required as lack of spacer 21 which is a characteristic specification of this close has also been detected in Iranian *M. bovis* isolates (SB0265) (35) (Fig. 6).

## Conclusions and future work

The very recent highly escalated political tensions seen in the international community over Iran's nuclear interests has resulted in imposing the most serious trade sanctions ever seen on Iran. Given the fact that the envisaged reductions in the country's oil revenues might continue for long in the coming years, cuts in funding of health schemes including test-and-slaughter program is likely. Working to develop a bovine Tb vaccine therefore, seems to be a very useful economical way to support the current bovine Tb control scheme in Iran.
